# Chemotherapy in Schwannomatosis: Justified Strategy or Deviation From Surgical Standard of Care?

**DOI:** 10.1002/ccr3.72953

**Published:** 2026-06-12

**Authors:** Muhammad Abdullah Awan

**Affiliations:** ^1^ Wah Medical College, National University of Medical Sciences Wah cantt Rawalpindi Pakistan

**Keywords:** chemotherapy, neurofibromatosis, peripheral nerve sheath tumor, schwannoma, schwannomatosis, surgical management

## Abstract

Although schwannomatosis is a benign peripheral nerve sheath disorder, management decisions become challenging in recurrent diffuse disease. Before systemic chemotherapy is initiated, careful distinction between benign schwannomatosis and malignant peripheral nerve sheath tumors is essential, as surgery remains the established standard of care.

Dear Editor,

1

I read with great interest the case report by Pedun et al. titled “A Rare Case of Diffuse Extra‐Cranial Schwannomatosis of the Trunk and Limbs,” published in Clinical Case Reports. The authors describe a rare and clinically important case of diffuse extra‐cranial schwannomatosis involving the trunk and limbs in a 44‐year‐old patient managed in a resource‐limited setting [1]. This report is valuable because it highlights the diagnostic importance of computed tomography and histopathology in differentiating schwannomatosis from other peripheral nerve sheath tumors.

One of the strongest clinical questions raised by this case is the decision to administer systemic chemotherapy despite histopathological confirmation of benign schwannomas. The patient received doxorubicin, ifosfamide, mesna, and steroids, with clinical regression of some palpable masses after several cycles. While this response is notable, schwannomatosis is generally considered a benign peripheral nerve sheath disorder, and surgical excision remains the usual standard treatment for symptomatic lesions [1].

Recurrence after previous surgery does not necessarily indicate malignant transformation. In recurrent schwannomatosis, tumor burden, anatomical location, surgical accessibility, pain, neurological compromise, and patient preference all influence management decisions. However, before cytotoxic therapy is considered, careful distinction between benign schwannomatosis and malignant peripheral nerve sheath tumor is essential. This is particularly important because systemic chemotherapy carries meaningful toxicity and its role in histologically benign schwannomatosis remains uncertain.

Another notable aspect of the case is the diagnostic limitation created by the absence of genetic testing and brain MRI. The authors appropriately mention that MRI is the preferred imaging modality and that genetic testing such as SMARCB1 or LZTR1 mutation analysis was not available. Although the clinical picture and histopathology supported schwannomatosis, incomplete exclusion of NF2‐related disease introduces some diagnostic uncertainty, which may influence long‐term counseling, surveillance, and treatment planning [1].

At the same time, this case also reflects the realities of resource‐limited practice. Extensive multifocal disease involving the axilla, mediastinum, pelvis, retroperitoneum, gluteal region, and inguinal region may make repeated surgical intervention difficult. In such circumstances, chemotherapy may be chosen as a pragmatic or palliative strategy when definitive surgery, MRI, genetic testing, or specialized multidisciplinary care are not readily accessible.

Importantly, this case raises a broader clinical question regarding therapeutic decision‐making in benign but diffuse schwannomatosis. Clinicians may benefit from clearer guidance on when non‐surgical treatment can be justified, how response should be monitored, and what threshold should prompt renewed suspicion of malignant change. Future reports should ideally document long‐term follow‐up, imaging response, symptom control, functional outcomes, and adverse effects when systemic therapy is used in benign schwannomatosis.

## 2 Conclusion

This case therefore provides a valuable reminder that diffuse schwannomatosis requires careful differentiation from NF1, NF2‐related disease, and malignant peripheral nerve sheath tumors before treatment planning. While chemotherapy may have been selected because of recurrence, disease burden, or resource limitations, its use in histologically benign schwannomatosis remains controversial. Increased clarity regarding surgical, palliative, and systemic treatment pathways may improve decision‐making in similar complex cases.

Sincerely,

Muhammad Abdullah Awan

## Author Contributions

**Muhammad Abdullah Awan:** conceptualization, formal analysis, writing – original draft, writing – review and editing.

## Funding

The author has nothing to report.

## Conflicts of Interest

The author declares no conflicts of interest.

## Data Availability

Data sharing not applicable to this article as no datasets were generated or analysed during the current study.
